# Psycho-social impacts, experiences and perspectives of patients with Cutaneous Leishmaniasis regarding treatment options and case management: An exploratory qualitative study in Tunisia

**DOI:** 10.1371/journal.pone.0242494

**Published:** 2020-12-01

**Authors:** Aicha Boukthir, Jihene Bettaieb, Astrid C. Erber, Hind Bouguerra, Rym Mallekh, Ikbel Naouar, Adel Gharbi, Manal Alghamdi, Emma Plugge, Piero Olliaro, Afif Ben Salah

**Affiliations:** 1 Department of Medical Epidemiology, Pasteur Institute of Tunis, Tunis, Tunisia; 2 Laboratory of Transmission, Control and Immunobiology of Infections (LR11IPT02), Pasteur Institute of Tunis, Tunis, Tunisia; 3 Department of Epidemiology, Center for Public Health, Medical University of Vienna, Vienna, Austria; 4 Nuffield Department of Medicine, Centre for Tropical Medicine and Global Health, University of Oxford, Oxford, United Kingdom; 5 Observatoire National des Maladies Nouvelles et Emergentes, Tunis, Tunisia; 6 Department of Family and Community Medicine, College of Medicine and Medical Sciences, Arabian Gulf University, Manama, Bahrain; 7 UK Collaborating Centre for the WHO Health in Prisons Programme, Public Health England, Reading, United Kingdom; 8 Special Program for Research & Training in Tropical Diseases (WHO/TDR), Geneva, Switzerland; ESIC Medical College & PGIMSR, INDIA

## Abstract

Although non-fatal and mostly self-healing in the case of *Leishmania (L*.*) major*, cutaneous leishmaniasis (CL) is mainly treated to reduce lesion healing time. Less attention is paid to the improvement of scars, especially in aesthetically relevant areas of the body, which can dramatically affect patients’ wellbeing. We explored patients’ perspectives about treatment options and the social and psychological burden of disease (lesion and scar). Individual in-depth interviews were conducted with ten confirmed CL patients at two *L*. *major* endemic sites in Southern Tunisia (Sidi Bouzid and Gafsa). Participants were selected using a sampling approach along a spectrum covering e.g. age, sex, and clinical presentation. Patients’ experiences, opinions and preferences were explored, and their detailed accounts gave an insight on the impact of CL on their everyday lives. The impact of CL was found to be considerable. Most patients were not satisfied with treatment performance and case management. They expected a shorter healing time and better accessibility of the health system. Tolerance of the burden of disease was variable and ranged from acceptance of hidden scars to suicidal thoughts resulting from the fear to become handicapped, and the stress caused by close relatives. Some believed CL to be a form of skin cancer. Unexpectedly, this finding shows the big gap between the perspectives of patients and assumptions of health professionals regarding this disease. This study provided valuable information for better case management emphasizing the importance of improving communication with patients, and accessibility to treatment. It generated context-specific knowledge to policy makers in Tunisia to implement effective case management in a country where access to treatment remains a challenge due to socio-economic and geographic barriers despite a long tradition in CL control.

## Introduction

Leishmaniasis is a vector-borne disease caused by a protozoan parasite of the species *Leishmania* and transmitted by the bite of infected sand flies (female phlebotomine). Cutaneous Leishmaniasis (CL) is the most common of the three forms of Leishmaniasis with an estimation of 0.7 to 1.2 million new CL cases occur worldwide annually [1]. Although it is widespread, African and Middle Eastern countries carry the major burden of CL [2].

In Tunisia, CL continues to be a significant public health concern due to its high incidence ranging from 2000 to 10 000 cases per year [3]. It is endemic in rural areas in several parts of the country, particularly in the central and southern regions with possible seasonal epidemics [3, 4].

The typical clinical manifestation of CL is painless skin ulceration on exposed parts of the body such as the face, hands and feet. This ulceration, which can be extensive and at multiple sites, heals slowly and spontaneously, but might leave permanent scars. Meglumine antimoniate remains the standard of care for extensive lesions of cutaneous leishmaniasis. Dosage and route of administration depend on clinical presentation, location and number of the lesions. Intralesional infiltrations with Meglumine antimoniate are used for patients with less than 5 and/or limb-localized lesions, while intramuscular injections are indicated for more severe lesions. Despite its satisfactory efficacy, Meglumine antimoniate can cause unpleasant side effects which may affect adherence to treatment. Cryotherapy, thermotherapy and local disinfection may be alternatives to antimonial drugs as recommended by the guidelines of the Tunisian Ministry of Health and the guidelines of the Eastern Mediterranean Office of WHO [5].

Although CL lesions are usually rather small and spontaneously curable, the remaining scars can be responsible for severe disfigurement and lifelong stigma. Patients are often psychologically and socially affected [5]. Women may face particular stigma because of marriage issues, especially in regions where their identity is based on conjugal and family life. While control strategies of CL have been widely implemented at the community level, this aspect has been largely neglected so far. Recently, the interest in the psychological impact of CL on patients and their quality of life has increased among researchers. Stigmatization and disfigurement were shown to be responsible for reduced self-esteem and even psychiatric problems such as anxiety and depression [6, 7].

A Tunisian study has examined the topic which revealed similar results regarding psychosocial effects of CL scars among patients from Sidi Bouzid [8]. This study was quantitative in nature, focused on women only and did not address the preferences of patients regarding case management alternatives and criteria of drugs’ effectiveness from their perspectives. Our present study attempts to fill these gaps using a qualitative approach.

Specifically, it aimed to understand the impact of CL lesions and scars on the everyday life of patients of both genders, in all its psychological, social and professional dimensions as well as their perspectives regarding treatment (efficacy, availability and preferences).

Although part of, and following the master protocol of, an international multicenter study as described in Erber et al. [9], it provided a more detailed analysis of specific needs of the Tunisian community in terms of prevention and treatment. Findings will help policy makers in Tunisia, to adapt case management especially by integrating expected psychological and social support in the treatment package of patients.

## Materials and methods

### Study setting

**Fig 1:**
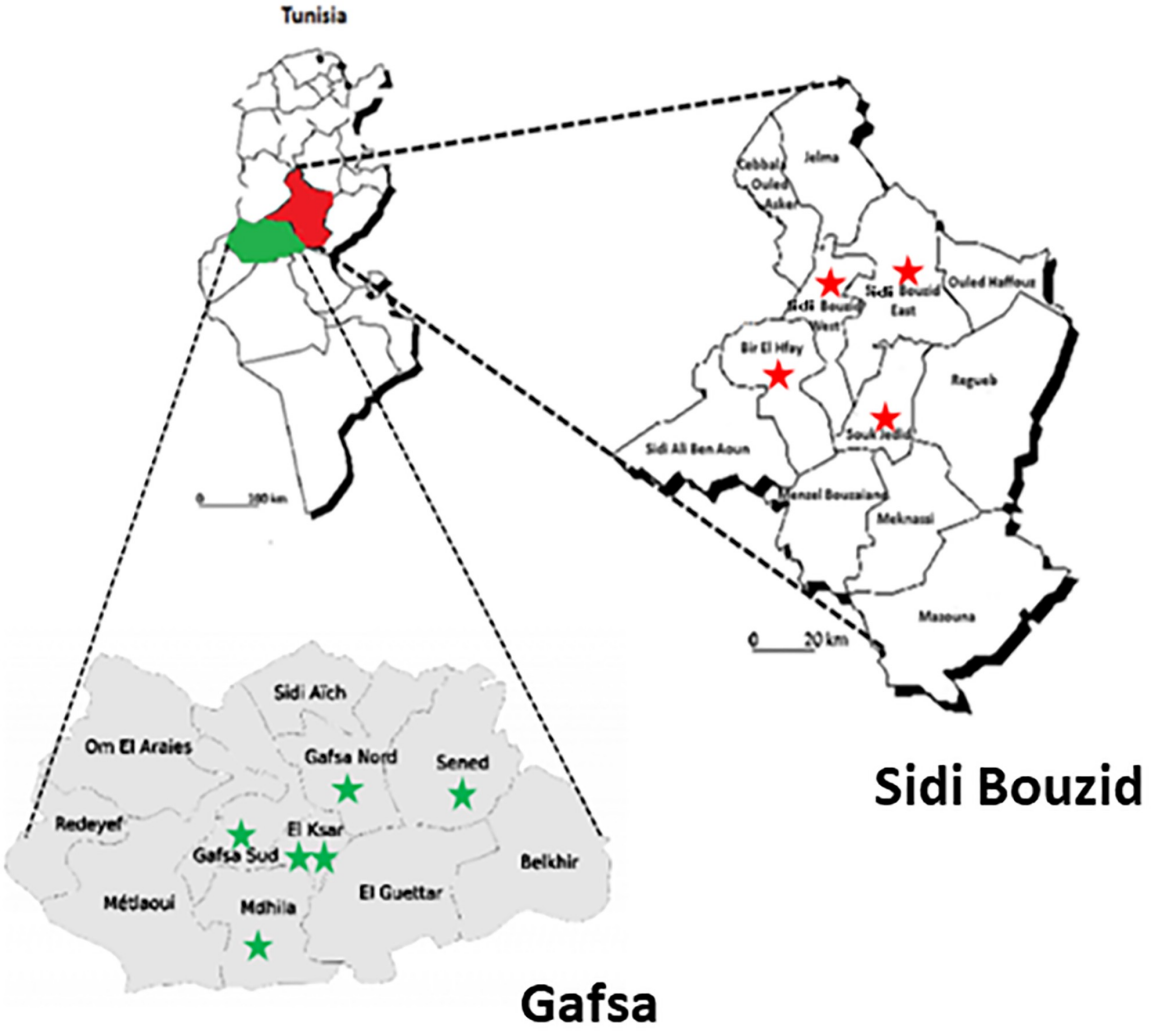
Location of Gafsa and Sidi Bouzid governorates in Tunisia.

Sidi Bouzid and Gafsa are two neighboring governorates located in central Tunisia (Fig 1), covering an area of 7807 and 7405 km^2^ and counting a total population of 445478 and 347225, respectively, according to the 2017 census [10].

The incidence of CL has been exceeding 1000 cases/100 000 persons per year in both governorates [11]. Combined with the cases from Kairouan, another central governorate, Sidi Bouzid and Gafsa account for the majority of CL cases reported across the country [3].

### Study design

This qualitative study was based on individual, semi-structured in-depth interviews conducted with ten CL patients in Sidi Bouzid and Gafsa, Tunisia. It was part of a large international multicenter study reporting patient-preferred treatment outcomes, where our group was highly involved [9, 12].

Although related, this Tunisian study therefore differs in its objectives, its analysis methodology, and therefore, its findings and implications, from Erber et al. (2020) [12]. The data (transcripts and notes) obtained were analyzed independently, using a coding framework developed according to its specific aims and objectives, and a thematic analysis approach based on these coded data (further detailed in the section Data analysis).

### Study participants and sampling

We used a purposive sampling method to ensure a maximum variation of information-rich sample, specific to the Tunisian CL form and context, to capture a broad spectrum of patients’ situations and experiences. This technique enabled us in collaboration with local HCWs to identify and select patients that were the most experienced with the phenomenon of interest (or with CL disease) [13]. It was important to ensure their availability and voluntary willingness to participate. The number of patients interviewed in depth (10) is not untypical for a qualitative study. While we would have liked to include a larger number, we were restricted by resource constraints, as outlined in Study participants and Sampling.

### Data collection

Interviews were conducted by a female epidemiologist (AB) who was introduced to the study area and trained by the principal investigator (ABS), who has a thorough experience in the epidemiology and the control of CL in the local and international context. Additional training and guidance were provided by an experienced qualitative researcher (EP), and support by the research team for analysis.

Interviews with duration of about one hour took place in the healthcare facility, except for one patient who was interviewed at home. All interviews were audio recorded and detailed notes taken before, during and after the interview. The interviews were conducted in Arabic, transcribed verbatim, and translated into English.

### Interview methodology

We used an adapted translated version of interview topic guide developed in collaboration of all investigators participating in the larger study [9] and validated by local teams. It included a set of open-ended questions which covered all study objectives S1 File. This approach encouraged respondent to express freely their views. the interviewer reformulated or clarified the questions probed to further explore the topics brought by the respondents. Newly emerged questions fed the interview guide when needed, to insure deepness in information an neutral understanding of patients’ perspectives around the study objectives. Before the interview, and following the written informed consent, we collected information on patients such as the age, gender, profession, residence (rural/urban area) as well as the number of lesions/scars and the treatment prescribed (type and location of health care).

### Data analysis

After reading the transcripts twice, individual sections were initially organized into an appropriate coding framework using NVivo^®^ 11, which was subsequently modified to accommodate the data (Table 1). In a second step, themes (as described in Green & Thorogood and Ziebland & McPherson [14, 15]) were developed from the coded data by two trained researchers (AB and ACE). Printouts of transcript sections that were coded to relevant nodes were manually arranged into emerging key themes on a single sheet of paper, and consensus achieved through discussion.

**Table 1:** The coding framework used.

**Patients experiences with the disease from their perspectives** Beliefs Causes Transmission Fears Treatment Prevention	**Treatment** Experience/ Seeking treatment Types Knowledge about Efficacy Duration Side effects Patient feelings about: Treatment in general, traditional/medical/self-treatments Time of healing Barriers Geographical Financial Cultural Follow up/ Completion Satisfaction / Preferences Recommendation Administration Pills/IV/D Duration of treatment Inconveniences	**Recommendations /Messages** to policy makers To stakeholders to CL new affected patients to doctors
**Psychosocial impact** Social stigmatization Cosmetic effects/scars Disease complications Disease relapse/reinfection Healing process **Impact on quality of life** Sleep Limitations in everyday life and disability Work		

Nodes are in bold.

Patients’ socio-demographic data were entered in Excel sheet and we calculated means and medians to analyze age and number of lesions and scars.

### Ethical considerations

The protocol including supporting documents (Patient information sheet, Informed consent declaration, Interview topic guide and Statement of compliance and confidentiality), received ethical clearance from the World Health Organization Research Ethics Review Committee (WHO ERC), Geneva, Switzerland and the Comité d'éthique Biomédicale de l’Institut Pasteur de Tunis, Tunis, Tunisia.

The consent procedure followed the protocol and all IRB requirements. Investigators took all safeguards to avoid coercion of patients, by emphasizing the voluntary informed consent and that medical services were independent of their enrolment in the study. An appropriate ID replaced patients’ names during transcription, and their identity was not disclosed at any time. The study was authorized by the Ministry of Health of Tunisia at the central (National Program of Control) and regional (Regional Directorate of Health of Gafsa and Sidi Bouzid) levels.

## Results

### Characteristics of study participants

We conducted interviews with ten CL patients (four men and six women) (Table 2). Age ranged between 27 and 65 years with an average of 39.1 years. All patients were confirmed to be infected with *L*. *major* parasites. The number of lesions/scars the patients suffered from was 1 to 5 with medians equal to 1 and 2, respectively. Patients were treated with Antimoniate of meglumine, Metronidazole, cryotherapy or local treatment. Four were treated at the primary health care center of Sidi Bouzid, five had more severe lesions and were treated in the regional hospital of Gafsa and one patient sought care in the private sector.

**Table 2:** Description of study participants.

Patient	Gender	Age	Profession	Education level	Current treatment	Marital status	Residence area	Lesion	Scar	Lesion/scar location
**1**	Male	32	Driver	License degree	Cryotherapy	Single	Rural	1	1	Ankle/shoulder
**2**	Male	65	Farmer	Unschooled	Antimoniate of meglumine	Married	Rural	5		Ankle
					Pills, Betadine					
**3**	Woman	27	Jobless	Bachelor degree	Cryotherapy, Metronidazole and self-medication	Single	Urban	1 (severe)		Leg/Shin
**4**	Woman	32	Farmer	Elementary education	Metronidazole, antibiotics & traditional remedies	Married	Rural	1 (severe)		Leg/Shin
**5**	Male	54	Teacher	License degree	Metronidazole, antibiotics, Local treatment	Divorced	Urban	1		Hand
**6**	Male	37	Guard National Officer	Bachelor degree	Antimoniate of meglumine, Metronidazole, antibiotics Cryotherapy	Married	Urban	5		Leg/ Ankle
**7**	Woman	28	Jobless	License degree	Cryotherapy, local treatment	Single	Urban	2	2	Leg
**8**	Woman	38	Agriculture worker	Elementary education	Cryotherapy	Single	Rural		2	Leg/hand
**9**	Woman	42	Jobless	Unschooled	Local treatment	Single	Rural		4	Face
**10**	Woman	36	Jobless	Elementary education	Not yet treated	Married	Rural	1 (severe)		Arm

**Fig 2:**
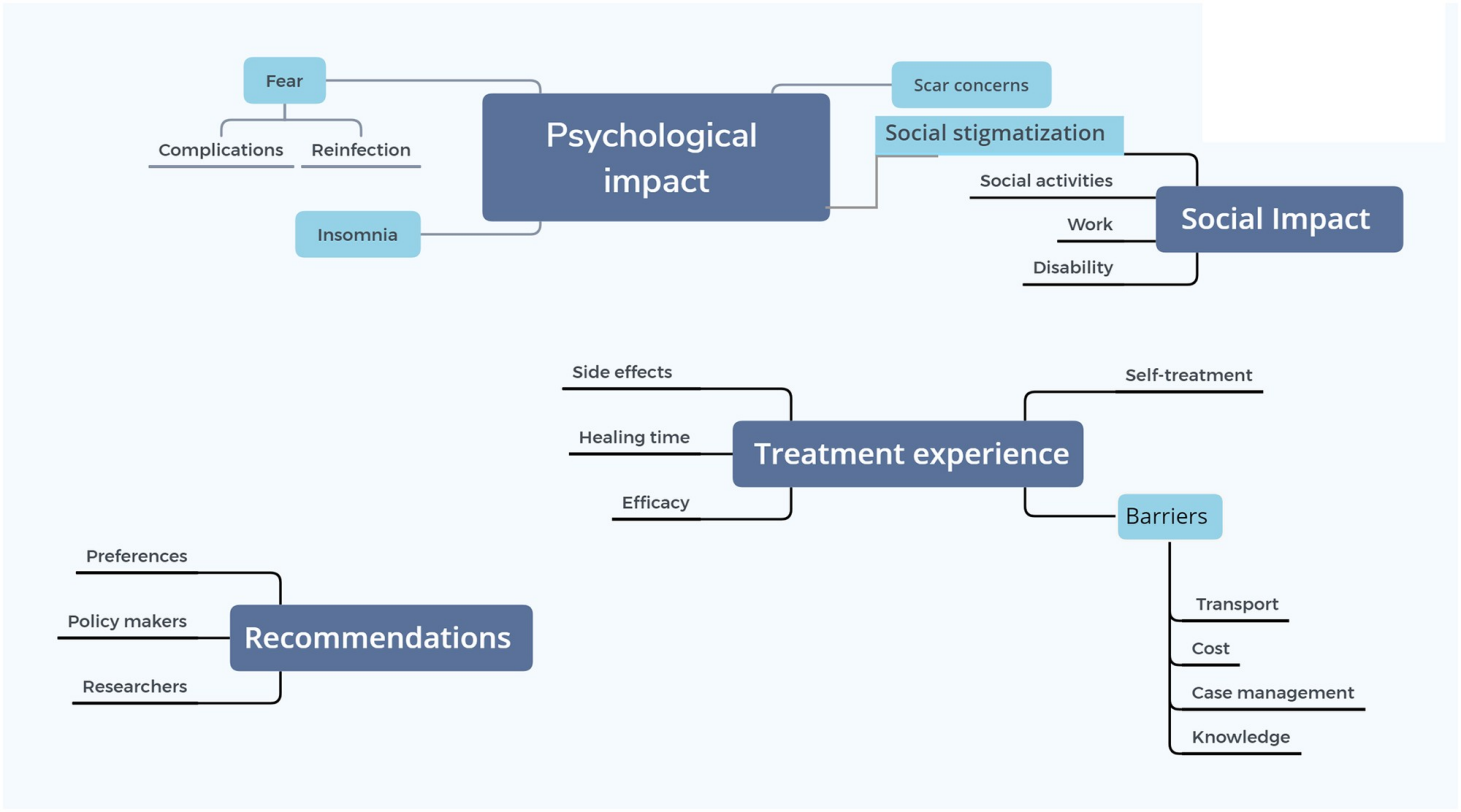
Main study themes and subthemes derived from the in-depth interview transcripts.

The main topics analyzed were how CL patients felt about their lesions or scars, what were their experiences of the medical care and treatment received and if they had any recommendations to improve that (Fig 2).

### Psychosocial impact of the disease

Overall, the social and psychological impact on patients was significant.

‘*It’s a catastrophe it is like a curse*!’

was the appeal of a 28-year-old young woman.

Patients’ fears were related to social stigmatization, often connected to the scar formation, reinfection or disease complications such as mutilation or amputation.

#### Social stigmatization

Social stigmatization was frequently linked to the appearance of the lesions. A 54-year-old teacher was worried about people’s response. He declared to be exhausted by his overwhelming apprehension of being rejected by society. His fears turned out to be valid when he noticed that some people avoided shaking hands with him as he had an active lesion on his right hand. He pointed out their misconception of the mode of transmission of the disease, as they believed it was contagious.

‘*The most bothering is the fear of people of this disease; they are scared to be contaminated because they don’t know it. Some of them walk away from me especially when the sore was infected it has an ugly look. Other people watch your sore with pity. You feel that you have a strange thing*.’

Likewise, a young woman aged 38 years was concerned about her colleagues’ possible reaction. They might move away and avoid sharing meals with her. She, herself, described her scars as “ugly” and as a possible source of disgust.

#### Concerns raised by the lesion/scar

The scar was also regarded as a potential source of rejection and stigma, hence the fear of its persistence.

*‘I was shocked*, *of the lesion; (…) The most frightening is that it remains’* stated a young woman.’

Patients perceived the scar as problematic, as it would remain. Plastic surgery was brought up as an option by several patients but was considered expensive. A young female job-seeker with a scar on her leg mentioned that if the scar were on her face, it could prevent her from finding a job.

This finding highlights gender-specific issues raised by the lesion (whether active lesion or healed scar). Patients perceived women as more affected than men when it comes to aesthetic aspects:

‘*If it’s on the face, the woman gets depressed… She will look for cosmetic surgery*.’

#### Fear of disease complications

Several patients believed that CL could affect deep tissues and eventually lead to amputation, although this was not an actual risk, except in case of comorbidities like diabetes.

‘*I am desperate, everyone can see that my sore opened its mouth. it’s over because my sore is very bad. I am convinced that my leg will be cut…I will kill myself, at the same time I am thinking about my little daughter, but I can’t live without my leg*.’

cried out a young diabetic mother, with a severe sore. She was afraid that CL could lead to the amputation of her leg and she even had suicidal thoughts, but at the same time, she was concerned about her daughter’s future life without her, and excluded the suicide option.

Another young woman felt that the disease was eating her flesh quickly:

*‘Nurses and doctors were shocked when they saw my sore*. *They asked me how I got it and how it ate my leg flesh*. *It is like skin cancer for me*, *because it ate my flesh in a short time*. *When I woke up I noticed that it has enlarged and when I put something on it*, *it absorbed it immediately*. *Perhaps if I didn’t care about it*, *the tissue could be eaten to the bone*.’

The patient likened CL to cancer. Every day she woke up feeling that her sore is enlarging. She looked visibly distressed while talking about her disease.

#### Fear of future reinfection

Some patients feared the eventuality to get the infection again next season, as in this patient’s case:

‘*I can be infected in other parts of my body, such as my face, next summer. What can we do to be protected from these infected flies?…I am thinking about my children*.’

They also asserted that they did not feel well informed regarding reinfection. They were concerned about their children and their relatives’ risk of infection by CL in the future.

‘*I’m scared of this disease. I have a phobia now*.’

#### Impact on quality of sleep

Thoughts about CL affected the patients’ quality of sleep. All of them complained about insomnia due to the fear of complications and due to the pain, which exacerbates at night. *A* young woman reported that she couldn’t cover her leg despite the cold because her sore was infected.

‘*When I cover my leg, I feel my sore scalding*.’

She felt she didn’t have another option than having to choose between freezing or extreme pain.

#### Limitations in everyday life and disability

Many patients reported being restricted to their bed for a certain period, unable to move and most patients complained about being limited in their everyday activities.

A man, 65 years old, reported:

‘*I couldn’t move from my bed. I went to the bathroom with a cane. I looked like a disabled person*.’

A young woman, 27 years old, explained that she couldn’t put on her clothes by herself. She had to go to the bathroom in a wheelchair, mentioning:

‘*When I put my feet on the floor the blood flows from my sore*.’

Patients in the region of Gafsa also complained about not being able to wash their sores with water. In fact, *some doctors warn the patients about wetting the lesions*.

‘*The most bothering is that you can’t wash the sore with water. That is what I can’t stand*!’

The feeling of dependence and lack of autonomy in their daily activities affected patients’ mood especially when they lack the support of their relatives when they need them.

#### Impact on work and activities

Patients felt that CL had an impact on their everyday activities, making them unable to perform certain tasks. This often affected their work, sometimes causing them to lose their daily income. Patients reported their professional life was affected by CL. This was the case for a teacher who reported:

‘*With the leishmaniasis sore I feel that I am disabled. I can’t perform exercises as required*!’

A female agricultural worker, aged 38 years, with sores on her right ankle and her hand, revealed:

‘*The sore didn’t prevent me from going to work but I suffered a lot especially in winter when I worked in the field, I crouched on my knees. I got very cold, my sore got red and inflated*.’

Despite her pain, she didn’t stop working. She also reported being unable to wash dishes or clothes, despite wearing gloves.

A young woman complained that she couldn’t be physically active as she was bound to her bed for three weeks with a pillow under her infected leg, the only position she could endure. She covered it with a packing case in order to protect it from the airflow, which caused her pain and might soil her sore. She couldn’t even go out to get some sun or practicing her social activities.

### Treatment experience and access to treatment

Regarding their experience of treatment, all patients complained about the long healing time linked to the low treatment efficiency.

Access remained challenging because of multiple economic, social and geographic barriers. The lack of health care skilled health care personnel and means of transport was considered as an obstacle to adequate access to treatment.

#### Lack of case management

A woman from a village in Sidi Bouzid governorate described how she sought advice at the local health care center in her village. Since symptoms persisted, she went to a private doctor at 40 km from her village, where she received unspecific medication without success. She then sought help at the local hospital, was subsequently referred to the regional hospital 65 km from her home. Her sores were unsuccessfully treated with a spray, but CL was eventually diagnosed there. She was referred to the regional hospital of Gafsa, 95 km from her home, where she was hospitalized and treated successfully as an inpatient.

Besides diagnostic delay, communication with healthcare professionals was described as difficult by a 65-year-old unschooled man:

‘*When you ask them about something, they answer “I don’t know”. They don’t want to give you any information*.’

#### Mobility and transport

Patients pointed out that there was no appropriate transport available (neither public nor private), and the transportation fees to reach the health facility were costly.

A 32-year-old woman expressed patients’ need for a close hospital as the main problem in rural areas. She underlined the advantages of living in the city because of reduced exposure to sand flies, and better treatment facilities.

An elderly man patient complained about the mode of transport, and how it affected his lesion:

‘*I can’t reach the hospital easily. I cannot walk, my son drives me there on his motorbike. However, my leg once got stuck in the motor bike, I couldn’t get off it*’

#### High costs of treatment in the private sector

Several patients decided to go seek private treatment despite the costs, as the general perception was that the private sector is more efficient. They reported that being treated in a public health care center requires a lot of patience.

‘*We think that being treated by a private dermatologist is better and more convenient than being treated in public sector*.*’*

In return, costs are high in the private sector whereas treatment for CL is provided for free in public hospitals and for patients covered by social insurance. Other products, e.g. disinfectants for home use, have to be bought. The private sector charges for both consultations and drugs. A male patient aged 37 stated:

‘*A little bottle of eosin costs 6 TND and a consultation costs 40 TND. Poor people can’t afford that*.’

A male patient who has chosen the private sector also mentioned:

“*There is no result except the pain and the expensive costs*.’

#### Treatment efficacy

Patients have different expectations with regards to their time of healing:

‘*I hoped to be healed since the first week I started the injections and red lotion application. First, my lesion got a little bit better, but after one or two weeks… there was no progress*.’

Most patients mentioned that neither Metronidazole pills nor local injections of Meglumine antimoniate worked. Many of them noticed that their lesions kept growing, which led some patients to try other options to heal their sores.

Almost all patients reported side effects linked to oral standard treatments (antibiotics), mainly digestive troubles and headaches. All patients found the intra-lesional injection (Meglumine antimoniate) made the lesion painful, sometimes more inflated and even disabling. A young man who had 5 lesions complained:

‘*After 2 injections, my sores got worse…I went to the bathroom crawling*.’

‘*The injections that I received in the lesions were painful, they didn’t work, my leg became more inflated*.’

The fear of painful and inefficient medical treatment associated to the economic factors, poor infrastructure and the lack of disease awareness, pushed CL patients to try chemical and herbal remedies and even uncommon practices to cure their sores. These self-treatments could be harmful for the sore. A patient, using urine, explains:

‘*When I applied the urines (on my lesion) it became wider*’

Sometimes, traditional treatments were used in combination with medical treatments, making the healing time longer.

‘*Each time I apply a new treatment on my lesion, it gets worse*.’

Although the first skin reaction when first time applied on the CL sore is frightening, the local cryotherapy treatment with liquid nitrogen was highly requested by many patients. They admitted that it’s the most effective of existing treatment for CL small lesions as it reduces the healing time and has better cosmetic results. A female patient, 28 years, explains:

‘*The pain of the cryotherapy, the mess after the treatment, the impurities and the liquid which get out of my sore, it stained my clothes and my bed. We have to stand that one to two weeks after that, it will get better*.’

‘*Honestly and according to my experience, the cryotherapy is the best treatment for leishmaniasis sore*.’

‘*The doctor cleaned my sore… she put on it “betadine” then treated it with cryotherapy, which healed it*.’

### Patients’ recommendations

Patients stated that CL disease could be better managed if medical treatment was sought at an early stage and advised against traditional treatments.

‘*We have to treat that disease at our earliest convenience to prevent its enlargement*.’

‘*I advise other patients to save themselves by going to the doctor early before the situation worsen*.’

A patient pointed out that the disease was benign, despite taking a long time to be cured. In contrast, most patients perceived CL as dangerous.

Overall, CL is perceived as a major public health problem associated with a significant stigma. Most patients complained about the lack or remoteness of health care facilities, emphasizing the need for a better access to treatment. Some patients asked to create healthcare units specialized in dermatology and plastic surgery in each regional hospital.

Patients also highlighted the necessity for environmental control in order to lower the disease burden. A young teacher suggested:

‘*They have to take care of the environment by cleaning dirty small lakes*.

They called for their right to obtain better information about the disease and suggested to authorities health education sessions about the disease *via* mass media, as well as prevention and control programs.

CL patients wonder whether researchers could improve therapeutic options so that treatment becomes more efficient and less painful. They also underlined the need for a vaccine to protect future generations.

‘*They have to invent a medication or a vaccine, as they did for influenza, a vaccine against this disease to avoid being infected by it*.’

‘*I am wondering scientists and researchers discovered treatments for serious diseases. Why can’t they discover a comfortable treatment for this disease*?’

Patients also suggested promoting CL patients’ non-governmental organizations to help them to understand and cope with the disease.

## Discussion

Our study is the first qualitative study to explore not only CL impact on Tunisian patients but also their perception and expectations of the treatment.

In addition to the core content of the master protocol described by Erber et al. (2018) [9], the present study addressed specific needs requested by the Ministry of Health of Tunisia and the ethics committee Pasteur Institute of Tunis. In fact, the interviews used for this study were conducted under a regional protocol, adapted to the context and including modifications as requested by the institutional review board, the “Comité d'éthique Biomédicale de l’Institut Pasteur de Tunis”, Tunisia. Our study shed light on Tunisian patients’ experience and knowledge of CL as a condition affecting their daily life, quality of work, quality of sleep and social interactions as well as their experience and perspectives regarding the treatment options, case management and care pathway in Tunisia. Our scope was to identify the specific needs of the community of Tunisian CL patients in order to guide the leaders of the National Program of CL control to fine tune health education, prevention and management plans. Whereas Erber and al.‘s focus [12] was on patient’s experience of the treatment of CL with the perspective of deriving patient-centered outcomes to be used as new endpoints to assess clinical trials.

Our findings explored the specific needs of the Tunisian population in a detailed approach, providing evidence derived directly from patients to policy makers and other stakeholders rather than clinical researchers. We achieved through this study a deep learning of this phenomenon from a new angle, we never used before during our 30 years of quantitative research on CL in Tunisia.

Unsurprisingly, women were more affected by the esthetic and disfigurement aspects of the scars. They felt more stigmatized and at higher risk to be affected in their prospects of marriage. Although a taboo issue, the fear of being rejected for marriage preoccupied affected young women. In these mostly rural regions, women’s role is focused around conjugal and family life making physical appearance very important. This gender-specific feature was also seen in Morocco [16], Afghanistan, Pakistan [17] and Yemen [18]. Affected women in all these regions are facing harsh social stigma and discrimination. Single women may find difficulties getting married while for wives, this may increase the stress in a marriage and might even lead to divorce.

Despite the fact that CL is a very old disease in the study area, the lack of knowledge of CL patients of their disease was a big issue, creating additional stress for the patients. Some patients considered that having a scar was like cancer or might lead to amputation. Other authors have also shown that most of the patients didn’t know exactly the disease symptoms and the mode of transmission and that many wrong beliefs were predominating [17, 19, 20]. These findings demonstrate the weakness of the National CL Control Program in providing proper health education contents and material and highlight the need to strengthen information and education about the disease, as well as about the risk of reinfection, since many patients expressed their concern about the spread in other parts of the body or the transmission of the infection to their relatives. This was similar to the Yemeni study where some affected mothers were trying to stay away from their children for fear of infecting them [18].

Although CL lesions are not supposed to cause a physical handicap, patients felt disabled and limited in their everyday life, by being restricted to bed or having difficulty to move, wear clothes or wash dishes. This impairment tended to exacerbate the anxiety and the psychological suffering caused by the scar. Professional life was also affected with higher absenteeism and limitation of many tasks at work. Similar results were reported by Al-Kamel where patients faced limited mobility and working capacity both inside and outside the home [18]. Undoubtedly, all these factors create a significant suffering and may lead to psychiatric disorders including anxiety, depression and even suicidal thoughts [7, 17]. Hence the need to consider psychological support as part of the case management in addition to early detection and medical care.

Our study also explored patients’ perception of the treatment and case management. The difficulty to reach health facilities, and how it affects patients’ physically and psychologically were striking. In fact, hospitals and health care centers are far to reach and no public transportation is available. All the possible transport is either expensive (private cars, taxis) or not appropriate such as bikes or motorcycles.

One patient’s words were very touching as she expressed that rural areas are not only the most affected by this disease, but they are also lacking proper treatment facilities. This was also reported in Morocco where rural areas lack access to health care [16].

More importantly, treatment efficacy was perceived as sub-optimal. Patients tried different treatments without any convincing results and often turned to traditional treatment. Side effects of treatment were reported in many cases and may lead to poor adherence and complications. An effective, user-friendly and affordable treatment, preferably in the form of ointment would be the best response to patients expressed needs [21–24]. Those treated with cryotherapy reported satisfactory results with better aesthetic results and shorter healing time. The promotion of such physical alternatives for CL treatment should be encouraged to give the patient and the physician more flexibility in treatment options.

We conducted this qualitative study rigorously and included a broad range of CL patients. It permitted to deeply understand patients’ needs and fears. The limitation of our study is mainly in relation to the small sample size because of resource constraints. However, the themes reported here were very rich and consistent across all participants.

## Conclusions

This is the first qualitative study in Tunisia addressing two important components of CL: its psychosocial impact on patients and their perception of the treatment. Our findings showed that this impact was significant in all the psychological, social and professional life aspects. Regarding treatment, patients were unhappy about its efficacy, the lack of standardized guidelines, and most importantly the lack of health care accessibility, which leads us to consider greater public health challenges in these endemic rural regions. This confirmed the limitation of the disease-centered management approach and supported a more patient-centered paradigm to meet the needs of the community.

Enhancing case management is crucial especially by involving pharmaceutical companies, academic investigators and other stakeholders towards new and more effective drug strategies complying with patients’ needs. Besides, psychological support should be included as part of CL treatment packages. For the time being, we believe that education and awareness campaigns at the community level can be a first step to reduce misconceptions about transmission cycle, stigma and social discrimination. To obtain a clearer picture of the real burden of CL and fine tune the control program, a future study with a larger sample size using mixed methods is planned. Findings of this study are passed to health authorities to be translated in appropriate policies meeting the patients’ needs and expectations. The frequently statement describing *L*. *major* CL as a benign disease in the scientific literature should be accepted from the perspective of disease-centered medical doctors only.

## Supporting information

S1 File The interview guide. (DOCX)
