# Supplementary material for: Psycho-social impacts, experiences and perspectives of patients with Cutaneous Leishmaniasis regarding treatment options and case management: An exploratory qualitative study in Tunisia
Source: PLoS One. 2020 Dec 1;15(12):e0242494. doi: 10.1371/journal.pone.0242494 (PMC7707605; doi:10.1371/journal.pone.0242494)
Supplement: S1 File — (DOCX) [file pone.0242494.s001.docx]

**S1 File. The Interview guide**

**Psycho-social impacts and treatment of cutaneous leishmaniasis: the perspectives of Tunisian patients**

1. **Knowledge attitude toward CL and treatment**
2. Can you start by telling me your story about CL?

*Seeking treatment, why now? Types of treatment, knowledge about treatments, efficacy of treatment, side effects, satisfaction with treatment, barriers to treatment (geographical, financial, cultural).*

1. What did you did do when you first found a lesion?
2. Tell me about the first time you noticed your lesion?
3. How did you feel when you noticed that you have CL?
4. How do you think you got it? *Do you know how to prevent it?*
5. What types of treatment did you look for?
6. What else did you try? *Medical, traditional, spiritual, self-treatment, what, when, where, why, who advised you?*
7. How the treatment works?
8. Can you tell me a bit more about the treatment?
9. How did/do you feel about the treatment?

*Cost, side effects, what happened, duration of treatment, number of cycles*

1. How easy was it to get treatment?
2. What happened after treatment?

*Follow up? Completion of treatment,*

1. Do you know any other treatments for CL?

*Other treatments that are not available?*

1. What do you think would be the best treatment for CL?

*Place of treatment, pills/IV/duration of treatment*

*What is/was most inconvenient in your current/past treatment?*

1. **EVERYDAY LIFE**

*Economic, social, emotional, psychological experiences, disability, limitations, work, relationships, effect on community, stigma, positive experiences, support, community,*

1. Can you describe your lesion?
2. How has the lesion changed your life? (alternative) What was your life like when you had CL?
3. How did people respond to your disease?

*Immediate family, friends, colleagues, community, health professionals*

*Positive and negative responses*

15. What help did you get?

*Who? What type of help? What helped you most?*

*Help before, during and after treatment*

1. Could you tell me how CL has affected you in your work?

*Modify depending on employment (careers, etc.)*

*Impact on salary/income if applicable*

1. Is there anything you can’t do or no longer do because of your disease?
2. **EXPECTATIONS/CONCERNS**

*What does cure mean to you? Feelings about treatments, cosmetic effects/scars, will I have a relapse, will I experience later complications*

1. How do you think your skin condition is going?
2. What did you hope for when you started the treatment?

*Treatment in general, traditional/medical/self-treatment*

1. What does healing mean to you?
2. Some people are concerned about scars. How do you feel about scars?

*Can you tell me a bit more about your scar? (if relevant)*

1. What do you think might happen in the future about your condition?

*Are you afraid that the skin condition come back (relapse/late complications)? Do you think being in treatment will prevent it* from coming back? Do you think you can prevent the scar with therapy?*

1. Some people believe CL can come back if you don’t get treatment. What do you think about this?
2. If you had the authority, what would you change for people with CL?

*Messages to others*

25. Finally, can I ask what does CL mean to you?

*Knowledge of CL, attitudes towards Cl.*
